# Discovery of Potential Plant-Derived Peptide Deformylase (PDF) Inhibitors for Multidrug-Resistant Bacteria Using Computational Studies

**DOI:** 10.3390/jcm7120563

**Published:** 2018-12-17

**Authors:** Shailima Rampogu, Amir Zeb, Ayoung Baek, Chanin Park, Minky Son, Keun Woo Lee

**Affiliations:** Division of Life Science, Division of Applied Life Science (BK21 Plus), Plant Molecular Biology and Biotechnology Research Center (PMBBRC), Gyeongsang National University (GNU), Jinju 52828, Korea; shailima.rampogu@gmail.com (S.R.); zebamir85@gmail.com (A.Z.); ayoung@gnu.ac.kr (A.B.); chaninpark0806@gmail.com (C.P.); minky@gnu.ac.kr (M.S.)

**Keywords:** multidrug-resistant bacteria, phytochemicals, dual pharmacophores, molecular dynamics (MD) simulation

## Abstract

Bacterial peptide deformylase (PDF) is an attractive target for developing novel inhibitors against several types of multidrug-resistant bacteria. The objective of the current study is to retrieve potential phytochemicals as prospective drugs against *Staphylococcus aureus* peptide deformylase (SaPDF). The current study focuses on applying ligand-based pharmacophore model (PharmL) and receptor-based pharmacophore (PharmR) approaches. Utilizing 20 known active compounds, pharmL was built and validated using Fischer’s randomization, test set method and the decoy set method. PharmR was generated from the knowledge imparted by the *Interaction Generation* protocol implemented on the Discovery Studio (DS) v4.5 and was validated using the decoy set that was employed for pharmL. The selection of pharmR was performed based upon the selectivity score and further utilizing the Pharmacophore Comparison module available on the DS. Subsequently, the validated pharmacophore models were escalated for Taiwan Indigenous Plants (TIP) database screening and furthermore, a drug-like evaluation was performed. Molecular docking was initiated for the resultant compounds, employing CDOCKER (available on the DS) and GOLD. Eventually, the stability of the final PDF–hit complexes was affirmed using molecular dynamics (MD) simulation conducted by GROMACS v5.0.6. The redeemed hits demonstrated a similar binding mode and stable intermolecular interactions with the key residues, as determined by no aberrant behaviour for 30 ns. Taken together, it can be stated that the hits can act as putative scaffolds against SaPDF, with a higher therapeutic value. Furthermore, they can act as fundamental structures for designing new drug candidates.

## 1. Introduction

Bacterial infections represent one of the major causes of death in humans [1]. One of the primary reasons for this is the capacity of microorganisms to develop resistance to existing antibiotics, thereby raising health concerns [2,3,4]. Currently existing antibiotics might develop resistance, posing a major challenge. There is thus a dire need for new antibiotics that can act on a broad spectrum of microorganisms.

Multidrug resistant (MDR) bacteria have often been described as a major impediment to public health globally [5] and are associated with nosocomial infections [6]. One notable reason for the increase in MDR bacteria is due to the unceasing administration of antimicrobial agents in pursuit of treating infections [7]. The bacterial species acquire resistance through several mechanisms such as by inducing mutations to alter the target protein [8,9,10], through enzymes involved in the inactivation of the antimicrobial agents (drugs) [11,12,13,14,15,16], by genes acquired from other species with low susceptibility to target proteins [17], and by avoiding the target [18,19,20,21].

Multidrug resistance can broadly be categorized into primary resistance and secondary resistance [7]. Primary resistance occurs when a drug confronts an organism for the first time, while acquired secondary resistance [22,23] is triggered in an organism after exposure to the drug, leading to intrinsic resistance or the extensive resistance. Intrinsic resistance refers to the lack of sensitivity of all the microorganisms of a single species to specific common first-line drugs [22] and is also known as multidrug resistance. On the contrary, extensive resistance (XDR) occurs when the microorganisms can withstand exposure to more than one potential antimicrobial agent [24]. These reports urge researchers to discover new targets and drugs that can effectively combat MDR bacteria.

Peptide deformylases (PDFs) are a class of metalloproteinase that are ubiquitously prevalent in microorganisms. This enzyme is present on the *def* gene and interestingly is different from the biochemical functions of the mammalian cells. Furthermore, developing potential antibiotics against this target induces the inhibitory effects against several organisms. Biologically, the PDF catalyses the deformylation step, essential for the biosynthesis and maturation of a protein. Bacterial protein synthesis requires the *N*-formylmethionine that is formed by enzymatic transformylation of methionyl-tRNA by formylmethionine tRNA transferase [25]. The nascent protein is changed to matured protein upon the removal of *N*-formyl methionine by a series of action by PDF. This cycle of formylation–deformylation is required for the growth of the bacteria and is seen in all the bacterial species [25].

Despite the presence of several PDF inhibitors, none of the potential inhibitors have been marketed [26,27]. Actinonin was one of the first antibiotics found to be potent against several bacteria, and remains as a prototype in developing the slow tight-binding type of inhibitors [26,28,29]. However, this compound was not considered for treatment [29] as the natural inhibitor lacks specificity [30] and triggers apoptosis [31,32,33]. Furthermore, it demonstrates minimal in vivo activity due to bacterial efflux [34,35] and might avoid the formylation pathway due to the endurance of resistance [36,37].

Bacterial PDFs can be categorized into PDF1 and PDF2, respectively depending upon their functions and their habitant. Type I PDFs are established in both Gram-negative and Gram-positive bacteria, while type II PDFs are confined in Gram-positive bacteria [38] and share a sequence identity of about 27~40% [1] with structurally conserved active site bearing a metal ion. Moreover, remarkable dissimilarities have been noticed towards the C-terminal regions of type I and type II PDFs. The C-terminal region of type I PDFs demonstrates α-helices, while in type II PDFs this region displays β-strands that are subsequently folded back onto themselves to form β-sheets.

Furthermore, PDF was also detected in humans, sharing a sequence identity of 28–34% with bacterial PDFs. Nevertheless, it was reported that the activity of PDF in normal human cells is quite low and is elevated in cancer cells [39]. Moreover, it was assumed that human mitochondrial PDFs might be non-functional or the antimicrobial agents might not reach the mitochondria due to the lack of appropriate evidence on toxicity [40,41]. Additionally, the mitochondrial S1’ subsite was revealed to be narrower than the bacterial PDFs, a trait which could be exploited in designing inhibitors against bacterial PDFs with relatively no effects on human PDF [29,42,43]. Taken together, PDF could be regarded as an excellent target for discovering novel antimicrobial agents against multidrug-resistant bacteria.

Since the ancient ages nature has been offering a stewardship to humankind by providing abundant sources of medicines [44]. Plant-derived compounds were foremost in demonstrating antimicrobial activity and hence have gained wider attention from the pharmaceutical and scientific communities [45,46,47]. Additionally, secondary metabolites of plants have been employed as therapeutic tools which exhibit varied ranges of activities enriched with several active compounds [48,49,50]. Besides being therapeutically active, plant phytochemicals induce low side effects and are abundant in availability, thereby being cost-effective [51,52]. Additionally, different phytochemicals have been proven effective against pathogenic multidrug-resistant bacteria [52,53,54,55,56,57,58,59,60]. Encouraged by these reports, the current investigation attempts to identify the potential phytochemical against PDF, employing combined ligand- and structure-based pharmacophore approaches along with molecular dynamics (MD) simulations.

## 2. Experimental Section

### 2.1. Ligand-Based Approach

#### 2.1.1. Dataset Construction and Its Composition

One of the preeminent criteria involved in the pharmacophore generation and subsequently its validation largely depends upon the compounds chosen. Specifically, the compounds should exhibit varying inhibitory activities (half maximal inhibitory concentration, IC_50_) with structural diversity. Furthermore, to obtain the most reliable pharmacophore model, a dataset of 51 compounds (https://www.bindingdb.org/bind/index.jsp) was grouped into the training set compounds and the test set compounds. The training set was employed to build the pharmacophore model, while the test set was adapted to validate the same. During the formation of the training set, care should be taken to include the most active compounds, and the set should encompass of a minimum of 16 compounds and should demonstrate 4–5 order magnitude of the activity data. Moreover, the dataset should be free of duplicates and any known inactive compounds. Herein, a total of 20 diverse compounds with different structures were assembled with the IC_50_ values ranging between 0.1 nmol/L and ~560,000 nmol/L. The test set compounds comprised of a total of 31 diverse structures with varied activity values. Careful selection of the test set compounds has been performed in order not to repeat the compounds of the training set. Correspondingly, the dataset was classified into most active, moderately active, and least active compounds based upon the inhibitory activity values. Accordingly, the compounds that exhibited inhibitory activity values less than 100 nmol/L (+++) were labelled as most active, the compounds with the inhibitory activity values existing between 100 nmol/L and ~10,000 nmol/L (++) were referred to as moderately active, and the compounds demonstrating the inhibitory activity values greater than 10,000 nmol/L (+) inhibitory activity values were regarded as most inactive compounds, respectively.

#### 2.1.2. Generation of the Pharmacophore Model

To generate the most efficient pharmacophore model, the structural features of the 20 training set compounds were exploited employing the *Feature Mapping* protocol available in the Discovery Studio (DS) (Accelrys Inc., San Diego, CA, USA). This module probes into the ligand’s structures and derives all the possible pharmacophore features imbibed by the ligands. The knowledge gained was invested in the selection of the features to obtain the suitable pharmacophore model. The *3D Quantitative Structure Activity Relationship (QSAR) Pharmacophore Generation* module, accessible on the DS, was initiated to secure a statistically significant pharmacophore model. Mechanistically, the 3D QSAR module depends on the *HypoGen* (available with DS) algorithm to glean the pharmacophore models from a given set of training set compounds. The generated pharmacophore model reflects the ability of the ligands to fit onto the pharmacophore. Furthermore, for the generation of the most dynamic pharmacophore model, properties such as activity and the uncertainty values for the training set compounds (input ligands) play a determinant role. For the current investigation, the IC_50_ value was considered as the activity property and an uncertainty property of 3 was chosen. The minimum and the maximum features were selected as 0 and 5, while the minimum feature points and the minimum subset points were set to 4 with weight variation of 0.302, respectively. The *Fas*t conformation generation, with a maximum of 10 pharmacophores and a minimum interfeature distance of 2.97, was opted for. From the resultant pharmacophore models, the ideal model was chosen based upon Debnath’s method. Accordingly, a significant model should portray low cost value, high cost difference with low root-mean-square deviation (RMSD) and high correlation.

### 2.2. Generation of the Receptor-Based Pharmacophore Model

Receptor-based pharmacophore model generation takes into consideration the inbound cocrystal that imparts knowledge on the key residues useful for inhibition. For the present study, the protein structure (PDB code: 1Q1Y) of peptide deformylase from *Staphylococcus aureus* with an innate ligand actinonin was employed. During this process, the information pertaining to all the available crystal structures was studied (UniProtKB-P68826). To logically probe into the pharmacophore features located in proximity with the innate ligand around 7 Å, the *Interaction Generation* module available with the DS was applied. The *Receptor–Ligand Pharmacophore Generation* module embedded with DS was launched to obtain the pharmacophore models that are complementary to the active site key residues. The generated pharmacophore model was obtained from the features that correspond to the protein–ligand interactions and are evaluated from the features. The best pharmacophore model was selected based upon the highest selectivity as predicted by the genetic function approximation (GFA).

### 2.3. Validation of the Pharmacophore Models

The selected pharmacophores from both the approaches were subsequently validated to assess their robustness in predicting the activities and redeeming the active compounds. Subsequently, the ligand-based pharmacophore (hereinafter pharmL) was validated by Fischer’s randomization method and the test set method, while receptor based pharmacophore model (hereinafter pharmR) was validated by receiver operating characteristic (ROC) plot analysis. Furthermore, in order to ensure the ability of both the pharmacophores in retrieving the compounds from the same database, a common validation method was conducted called as the decoy set validation method.

#### 2.3.1. Ligand-Based Pharmacophore Model Validation

The obtained pharmacophore model should be statistically significant and should possess the ability of accurately retrieving the active compounds thereby predicting their activities. Accordingly, the best pharmacophore model that has obeyed the Debnath’s analysis was subjected to Fischer’s randomization and test set methods of validations. Fischer’s randomization critically acknowledges that the pharmacophore model was not generated arbitrary which is reflected by the low cost values. Fischer’s randomization was executed alongside the pharmacophore generation at a statistical significance of 95% computed by the formula

(1) $$S=1-\left(1+\frac{X}{Y}\right)100$$

where X denotes the total number of hypothesis with a cost value typically lower than the significant hypothesis and Y indicates the number of initial and the random HypoGen runs. Correspondingly, 19 random spreadsheets were generated by random shuffling of the activities of the training set compounds.

The test set method of validation evaluates the ability of the chosen pharmacophore in categorizing the compounds other than the training set in the same order of magnitude as the experimentally obtained IC_50_ values. This method guides us to comprehend the ability of the pharmacophore model in identifying the active compounds.

#### 2.3.2. Receptor-Based Pharmacophore Model Validation

The pharmacophore model with high selectivity was subjected to validation alongside the pharmacophore generation opting the “validation” as true and the results were read as the ROC plots. The obtained plots were an objective and quantitative measure, and the adequacy of the chosen pharmacophore in distinguishing between the active and the inactive ligands. A graph was plotted with specificity on the x-axis and the sensitivity on the y-axis. If the model cannot discriminate between the active and the inactive compounds, the graph appears to be a straight line; however, upon gaining accuracy, the propensity of the curve tends towards the ideal condition where the sensitivity and the specificity will be one. The area under the curve (AUC) defines the accuracy and ranges between 0.5 (random) to 1.0 (excellent). For the current study, a total of 15 active compounds and 20 inactive compounds have been considered. Furthermore, the quality of the model was evaluated based upon the true positives, true negatives, false positives and false negatives, which determine the sensitivity and the specificity of the model. Sensitivity defines the ability of the pharmacophore model in determining the true positives and is computed employing the formula; sensitivity = TP/(TP + FN). In the equation, TP refers to true positives, and FN implies false negatives. Conversely, the term specificity denotes the ability of the model in identifying the negatives and is calculated by specificity = TN/(TN + FP), where TN refers to true negatives and FP represents false negatives, respectively.

#### 2.3.3. Decoy Set Method of Validation

The decoy set method of validation was implemented to substantiate the competence of pharmL and pharmR in retrieving the active compounds when subjected to screen an external database. In this pursuit a dataset (D) of 1000 compounds was instituted with 20 active compounds (A). Correspondingly, pharmL and pharmR were allowed to screen the database employing the *Ligand Pharmacophore Mapping* accessible with the DS using the Best algorithm. The subsequent results generated were assessed based upon the enrichment factor (EF) and the goodness of fit (GF) values and were enumerated utilizing the formulae

(2) $$\mathrm{EF}=\frac{\mathrm{Ha}\;\times \;D}{\mathrm{Ht}\;\times \;A}$$

(3) $$\mathrm{GF}=\left(\frac{\mathrm{Ha}}{4\mathrm{HtA}}\right)\left(3A+\mathrm{Ht}\right)\;\times \;\left\{1-\frac{\mathrm{Ht}-\mathrm{Ha}}{D-A}\right\}$$

### 2.4. Virtual Screening of the TIP Database

The validated pharmacophore was then allowed to screen the Taiwan Indigenous Plants (TIP) database [61,62,63]. This database is enriched with biologically active phytochemicals with anticancer, antiplatelet, and antituberculosis activities, as evidenced by the literature. Since nature has been an enormous source of medicines from the ancient ages, the present investigation makes an effort to retrieve potential candidate chemical compounds from the plant sources encompassed with the TIP database. Plant-derived natural compounds offer a host of beneficial features over synthetic medicines, such as low toxicity [64,65], fewer side effects, and abundance. The pharmL and pharmR are used as 3D query to screen 5284 chemical compounds furnished within the database employing the *Ligand Pharmacophore Mapping* with *Fast/Rigid* fitting method. The compounds mapped with both the models, implying that they carry the chemical groups essential for inhibition. Furthermore, these compounds were monitored for their drug-like properties employing the Lipinski’s Rule of Five (Ro5) and absorption, distribution, metabolism, and excretion (ADMET) assessment obtainable with the DS.

### 2.5. Drug-Like Assessment

To evaluate the ability of a drug for its good pharmacokinetics, the mapped compounds were escalated to delineate on their drug-like assessment. This approach helps in weeding out the non-drug like compounds from being processed further. Furthermore, such examinations establish the compounds as prospective drugs and enhances their developmental chances during the drug development pipeline. Accordingly, the *ADMET Descriptors* accessible with the DS was launched that specifically monitors if a compound could cross the blood–brain barrier (BBB), its solubility, its absorption (HIA), and toxicity. Correspondingly, the upper limit for BBB, solubility, and the absorption were fixed at 3, 3, and 0, respectively. The resultant compounds were subjected to Ro5, which is by far the most influential measure in the preclinical drug development. The Ro5 establishes the quality of a lead compound to make it an orally active drug. Subsequently, a drug should possess a molecular weight less than 500 Da, have fewer than five hydrogen bond donors, fewer than 10 hydrogen bond acceptors, and 10 rotatable bonds, and a Log *p*-value of less than 5. To accomplish this, *Filter by Lipinski* embedded in the DS was initiated.

### 2.6. Molecular Docking Studies

The compounds that obeyed the aforementioned criteria were upgraded to the molecular docking studies employing the CDOCKER available with the DS. Molecular docking is an effective method that guides us to screen the compounds that accommodate well at the proteins active site and reveals an ideal binding mode of the small molecules. The CDOCKER programme facilities the refinement docking for numerous ligands with a single target protein. This grid-based docking method, utilizes CHARMm wherein the protein is held tight while the ligands were allowed to move. The results were obtained as -CDOCKER energy and -CDOCKER interaction energy, where the higher the values, the greater the favourable binding between the protein and the ligand.

To ensure the accuracy of docking calculations, Genetic Optimisation for Ligand Docking (GOLD) v5.2.2 (The Cambridge Crystallographic Data Centre, Cambridge, UK) was used. GOLD has been widely successful in the field of virtual screening, lead optimization and further identifying the most precise binding modes for the ligands and predominately operates by inducing receptor flexibility obtained by the side chain flexibility. For the current study, the GoldScore was used as the default scoring function while the ChemScore was adapted as a rescore function. Furthermore, the GoldScore is a sum of van der Waals energy, ligand torsion strain, H-bonding energy, and metal interaction. The ChemScore quantifies the total free energy variations associated with the ligand binding together with hydrophobic–hydrophobic contact area, ligand flexibility, hydrogen bonding, and metal interaction.

The target structure for the current study is the peptide deformylase from *Staphylococcus aureus* with the PDB code 1Q1Y. This enzyme belongs to the hydrolase family having a resolution of 1.9 Å complexed with the natural inhibitor actinonin and a zinc ion. Prior to docking, the protein was prepared by enabling the Clean Protein protocol available on the DS. All the heteroatoms were removed and the hydrogen atoms were incorporated by applying the CHARMm forcefield. The active site was designated to all the atoms within the range of 10 Å around the innate ligand. Furthermore, the histidine protonation state was oriented as observed in the crystal structure.

The procured lead-like candidates were thereafter docked into the proteins active site. Subsequently, 100 conformers for each ligand were allowed to generated while retaining all the other parameters as default. Following this, the ideal binding modes were retrieved from the largest cluster, which was examined thoroughly for the key residue interactions, and higher dock scores than reference, hereinafter the most active compound from the training set.

### 2.7. Molecular Dynamics Simulation Studies

Molecular dynamics simulation studies were executed to comprehend on the dynamic behaviour of the ligands at the proteins active site in order to ensure the obtained binding modes and further to affirm the stability of the complex. The selected protein ligand complexes from the docking studies were employed as the initial structures for the MD studies. GROningen MAchine for Chemical Simulations v5.0 (GROMACS, www.gromacs.org) [53] was recruited for studying the nature of the protein and the ligand utilizing an all-atom CHARMM27 forcefield [53,66]. Furthermore, the topologies of all the ligands were secured employing SwissParam. The simulations were performed in the dodecahedron water box solvated with TIP3P water model and the system was neutralized with the counter ions. The steepest descent algorithm was applied on the initial structures to escape the steric clashes and the unsuitable geometry, thereby relaxing the initial structures with 10,000 steps with a maximum force below 1000 kJ/mol. Following this, a dual step equilibration process was conducted with (constant number of particles, volume, and temperature) NVT and (constant number of particles, pressure and temperature) NPT, respectively. The NVT ensemble (constant number of particles, volume, and temperature) was used for the first equilibration step for 1 ns at 300 K with a V-rescale thermostat. The NPT ensemble (constant number of particles, pressure, and temperature) was employed for the second step of equilibration for 1 ns at 1 bar with a Parrinello-Rahman barostat [67]. The bond constraints were monitored by the SETTLE [68] and LINear Constraint Solver (LINCS) [69] algorithm. Particle Mesh Ewald (PME) [70] was employed to compute the long-range electrostatic interactions, while short-range interactions and van der Waals interactions were measured applying an upper limit of 9 Å and 14 Å, respectively. The equilibrated NPT ensemble was subjected to MD simulations for 30 ns [71] with periodic boundary condition. The obtained results were evaluated employing the DS and visual molecular dynamics (VMD) [72].

### 2.8. Novelty Assessment of the Compounds

To further examine the novelty of the obtained hits specific to PDF, the Tanimoto similarity search was conducted against all the experimentally available known inhibitors of PDF enabling the Find Similar Molecules by Fingerprints module available with the DS, employing the predefined ECFP_4 fingerprint property. The ECFP_4 fingerprint property computes minimum, maximum and averages similarities and measures of nearness to known inhibitors. The Tanimoto similarity measures are computed as SA/(SA + SB + SC), the number of “and” bits normalized by the number of “or” bits, where SA refers to the number of AND bits present in both the target and the reference, SB is defined as the number of bits in the target but not the reference, and SC reflects the number of bits in the reference but not the target. Alternatively, the search was also performed using the online search employing the ChemSpider (http://www.chemspider.com/Default.aspx).

## 3. Results

### 3.1. Generation of the Pharmacophore Model

#### 3.1.1. Ligand-Based Pharmacophore Generation

**Figure 1:**
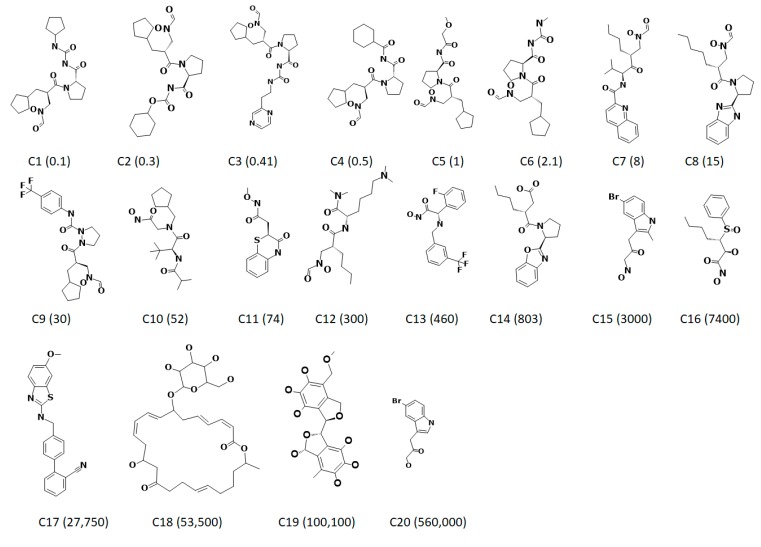
Two-dimensional structures of 20 training set compounds employed for the generation of ligand-based pharmacophore model. The experimentally determined half maximal inhibitory concentration (IC_50_) values are expressed in nmol/L in parenthesis.

**Figure 2:**
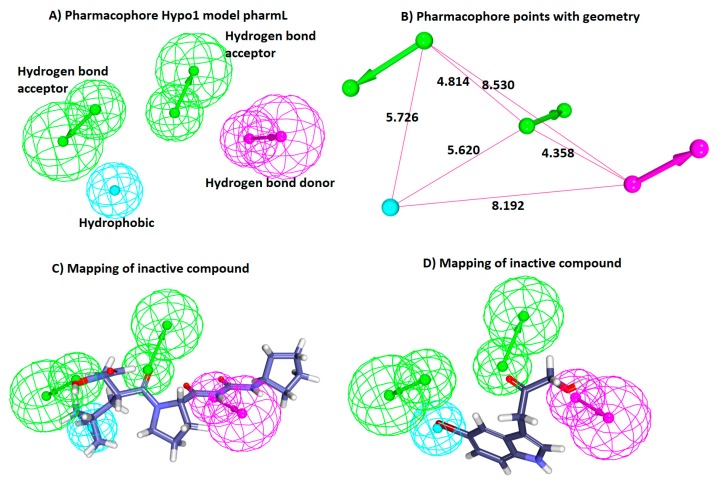
Characteristic four-featured HypoGen-guided pharmL. (**A**) PharmL consists of four features, including two hydrogen bond acceptor (HBA), one hydrogen bond donor (HBD), and one hydrophobic (HyP). (**B**) The geometry of the pharmL with its corresponding pharmacophore points. (**C**) Aligning of the most active compound to the model shows that it has mapped with all the features of pharmL. (**D**) Aligning of the inactive compound shows that it has mapped with three features of pharmL.

The ligand-based pharmacophore modelling was employed to exploit the key chemical features present on different known inhibitors crucial for inhibiting the target enzyme. In order to generate the statistically significant hypotheses, the HypoGen algorithm was employed that corresponds to the experimental and the predictive activities of the known inhibitors. Accordingly, the 20 known inhibitors (Figure 1) with divergent structures and IC_50_ values detected by the same bioassay methods have been considered. Guided by the results obtained from the *Feature Mapping* module, key features such as the hydrogen bond acceptor (HBA), hydrogen bond donor (HBD), hydrogen bond acceptor lipid (HBL), hydrophobic (HyP) and ring aromatic (RA) were considered during the pharmacophore generation. Subsequently, 10 hypotheses have been returned, utilizing the statistical parameters such as cost values, correlation, RMSD and the fit values (Table 1). Delineating on the hypotheses, it was revealed that all the hypotheses rendered HBA and HyP features as prompted by the *Feature Mapping* protocol. These findings led us to comprehend that the generated pharmacophore models have the essential features required for the inhibition of PDF. Correspondingly, to determine an ideal pharmacophore model, the analysis proceeds according to Debnath’s postulates, which state that a statistically significant model should display low total cost, high cost difference, low RMS and high correlation. The cost difference reports the obtained cost as a difference between the null and the total cost of the hypothesis. Subsequently, the probable difference if lies between 40 and 60 bits implies that the predictive correlation probability may exists between 70 and ~90%. Furthermore, if the difference is greater than 60 bits, it can be deduced that the propensity of the correlation probability might be greater than 90%. Hypo1 demonstrates a high cost difference of 113.10 illuminating its significance over the other hypotheses. Moreover, the correlation coefficient reflects the geometric fit index that was built on the linear regression. Hypo1 represented a high correlation coefficient of 0.90, portraying its favourable predictive ability. Additionally, the RMSD defines the variations of the predicted activity values from that of the experimental values. Hypo1 generated the lowest RMSD value when compared to all the hypotheses. Moreover, the cost values additionally govern the authenticity of the pharmacophore model by judging if the total cost value is far from the null cost and near to the fixed cost. In the current study, the null cost was computed to be 240.78 while the fixed was 86.77. Together, these results lead us to choose Hypo1 as it obeyed to the Debnath’s analysis. The preferred Hypo1, hereinafter referred to as pharmL, consists of four features, including two hydrogen bond acceptors, one hydrogen donor and one hydrophobic feature (Figure 2A,B).

Moreover, to determine the predictive potential of the pharmL, an evaluation of the inhibitory activities of the training set compounds using regression analysis was employed. PharmL efficiently estimated the activity values of the training set compounds in par with the experimental activities (Table 2). However, one active compound and one inactive compound were reported as moderately active compounds. These results determine the ability of Hypo1 in distinguishing the active compounds in a given dataset.

To determine the ability of pharmL in selecting the active compounds, the most active and the least active compound from the training set were subsequently superimposed. Upon superimposition, it vividly elucidated the accuracy of pharmL in distinguishing the active compounds from the inactive compounds. The most active compound with an IC_50_ value 0.1 nmol/L aligned with all the features of pharmL (Figure 2C), while the most inactive compound bearing the IC_50_ value 560,000 nmol/L mapped with three features (Figure 2D), thus showcasing the competence of pharmL in selecting the most active compounds upon subjecting it to screen the databases.

#### 3.1.2. Generation of Structure-Based Pharmacophore Generation

The structure-based pharmacophore modelling relies on the key features between the inbound ligand and the receptor to generate a pharmacophore model. For the current investigation, the crystal structure of *Staphylococcus aureus* (PDBcode: 1Q1Y) was employed. Furthermore, the well-defined active site groove demonstrates a remarkably conserved residues such as Val59, Gly60, Gln65, Leu112, and Glu155. Subsequently, enabling the *Receptor–Ligand Pharmacophore Generation* module resulted in 10 pharmacophore models with a maximum of six features and a selectivity score of 11.498 (Table 3).

**Figure 3:**
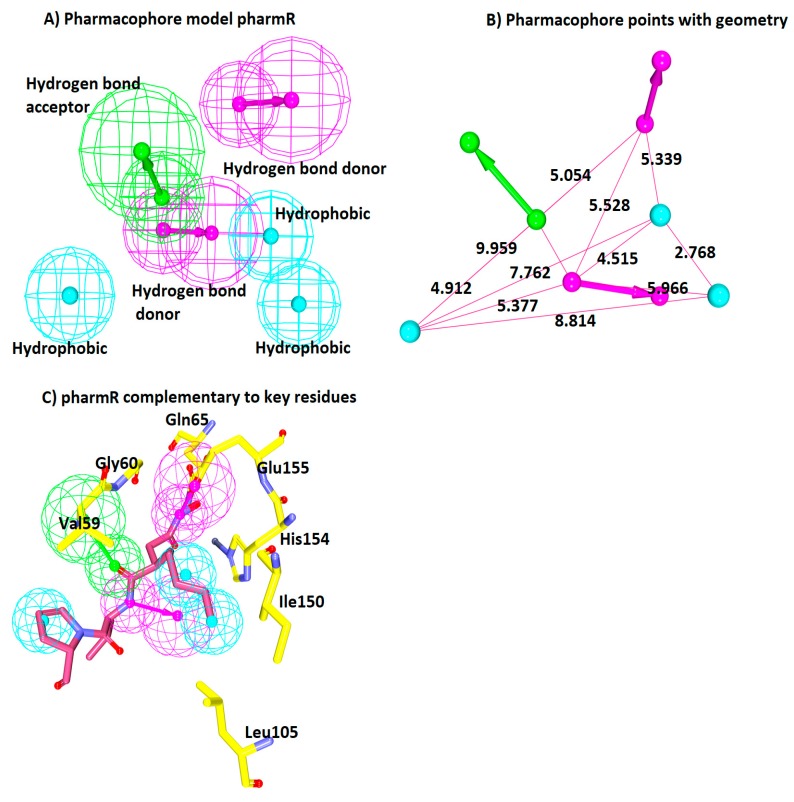
Receptor-based pharmacophore model generation. (**A**) A six-feature model pharmR consisting of three hydrophobic (HyP), two hydrogen bond donor (HBD), and one hydrogen bond acceptor (HBA). (**B**) Geometric interface distance between the features. (**C**) Key residues (yellow) complementary to the pharmacophore features. Pink line and stick represents the inbound ligand.

Furthermore, it was observed that the HBA and HyP features were present in all the models, showing their importance in the inhibition of the SaPDF. Moreover, these features were consistently observed in the models generated from the ligand-based pharmacophore approach (Table 3). From this, it can be deduced that the HBA and HyP features might be prominent in inducing the inhibitory mechanism. Furthermore, to select an ideal pharmacophore, the Pharmacophore Comparison module obtainable with the DS was employed. This step was conducted to secure the most reliable structure based model demonstrating the lowest RMSD with the ligand-based model. Correspondingly, the first model has rendered lower RMSD as compared to the other nine models and additionally has shown the complementarity against the catalytic active residues (Table 4 and Figure 3C). Therefore, this model was chosen and was labelled as pharmR. The pharmR contains six features, one HBA, two HBD, and three HyP features, which are complementary to the key residues. The pharmacophore features and the interfeature distance are represented in (Figure 3A,B). The six obtained features were complementary to the key residues. The HBA was complementary to the Val59 and Gly60 residues. One HBD was in the vicinity of Gln65 and Glu155. The two hydrophobic features were prompted from His154, Ile150, and Leu150. The third hydrophobic bond was noticed with the pentane ring of the ligand and the key residue Val59 (Figure 3C). 

### 3.2. Validation of the Pharmacophore Models

Validation of the generated pharmacophore models is a step that determines if the obtained models could retrieve the active chemical compounds upon subjecting them to database screening. Furthermore, pharmL was validated employing the Fischer’s randomization method and the test set method and pharmR were validated using the ROC. Additionally, to ensure the robustness of the pharmacophores a common method known as the decoy set method was conducted.

#### 3.2.1 Validation of PharmL

##### Fischer’s Randomization Method

Fischer’s randomization method was executed to judge the statistical significance of pharmL when a 95% confidence level was applied. Consequently, 19 random spreadsheets were generated by shuffling the experimental activity values to each training set compound. Upon comparing the cost values of the 19 random models with pharmL, it was revealed that the cost value of pharmL was far lower than the 19 hypothesis, implying that pharmL was not generated by chance and thereby illuminating its significance.

##### Test Set Method

Test set validation was performed to analyse the ability of the pharmacophore in categorizing the compounds in the same order of magnitude as in the activity scale. Accordingly, 31 compounds apart from the training set that exhibited diverse structures and varied IC_50_ values were considered. These compounds were grouped based upon their inhibitory activity values into three categories. The IC_50_ values less than 100 nmol/L (+++) were labelled as most active, the compounds with the inhibitory activity values existing between 100 nmol/L~10,000 nmol/L (++) were referred to as moderately active, and the compounds demonstrating the inhibitory activity values greater than 10,000 nmol/L (+) were called the inactive compounds, respectively. PharmL ably calculated the inhibitory activities of the compounds, however it misestimated two inactive compounds as moderately active (Table 5). Furthermore, the regression analysis demonstrated that pharmL displayed remarkable correlation coefficients between the experimental and predicted activities of the training and test set compounds of nearly 0.90. This result strengthen the ability of the model in differentiating the active compounds from the inactive compounds.

#### 3.2.2. Validation of PharmR

##### Receiver Operating Characteristic (ROC) Plot Analysis

**Figure 4:**
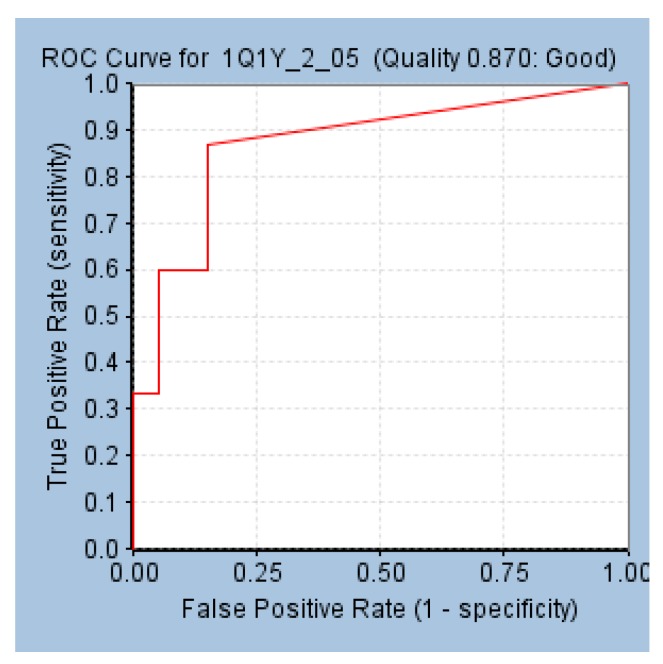
Validation of pharmR employing the receiver operating characteristic curve.

The receptor-based pharmacophore model, pharmR was validated employing the ROC by subsequently determining the area under the curve (AUC). A total of 35 compounds (15 known active + 20 known inactives) were considered alongside the model generation. As a result, the numbers of retrieved true positives and true negatives were 13 and 17, with false positives and false negatives being three and two, respectively. Furthermore, the sensitivity and the specificity were computed to be 0.86 and 0.85, respectively, with a model quality of 0.87. This score infers that the model is of a good quality and displays affinity towards the active compounds (Figure 4).

##### Decoy Set Method of Validation for PharmL and PharmR

The decoy set validation was performed for both the models to analysis their ability in redeeming the active compounds when subjected to an external dataset. An external dataset (D) of 1000 compounds was instituted with 20 active (A) compounds, and pharmL and pharmR were allowed to map, enabling the Ligand Pharmacophore Mapping protocol embedded with the DS with Fast flexible parameters, while retaining the other options as default. The robustness of the models was interpreted from the results obtained through enrichment factor (EF), and goodness of fit score (GF). PharmL has retrieved 25 hit, (total hits, Ht), compounds with 19 (Ha) active compounds, while PharmR mapped 24 (Ht) compounds that consisted of 20 (Ha) active compounds (Table 6). From the results, it can be observed that both the models exhibit an exceptional capability in inclining towards the active compounds as rendered by the percentage yield of the active compounds and the ratio of the actives being 95 % and 100 % for pharmL and pharmR, respectively (Table 6). The goodness of fit (GF) was adapted to judge the quality of the model that ranges between 0~1 implying a model to be null to ideal. From the results (Table 6) the GF values for pharmL and pharmR were computed as 0.79 and 0.83 respectively, reflecting the tendency of the models towards the true positives. These findings imply the usability of the models to screen databases in virtue of mapping to the prospective leads that can inhibit the PDF. From these findings it can be unequivocally stated that pharmL and pharmR are the quintessential models that can distinguish the active compounds from the inactive compounds.

### 3.3. Virtual Screening of Taiwan Indigenous Plants (TIP) Database

**Figure 5:**
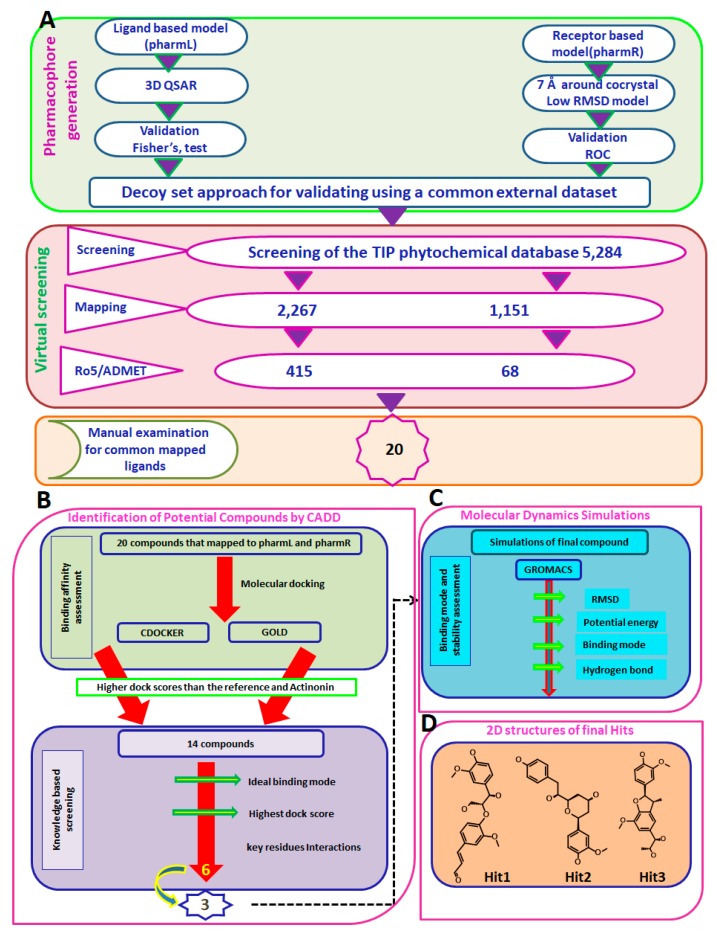
Computational methods to retrieve the potential candidate compounds. (**A**) Pictorial depiction of the generation of pharmacophore models and virtual screening process. (**B**) Sequential steps involved in binding affinities and knowledge based screening for potential compounds. (**C**) Evaluation of the binding modes and the stability of the final complex compounds through molecular simulations. (**D**) Two-dimensional representation of the potential compounds.

The TIP database was screened by pharmL and pharmR, which comprises the plant-derived chemical compounds (5284) that serve as an enriched source for biologically active phytochemicals. Subsequently, pharmL yielded 2267 compounds and pharmR resulted in 1151 compounds, respectively. Furthermore, their pharmacokinetic properties were determined and drug-like assessment was executed employing the ADMET Descriptors and Filter by Lipinski available in the DS, which retrieved 415 compounds by pharmL and 68 compounds by pharmR. The mapped compounds were manually visualized for the presence of common ligands that were procured from by pharmL and pharmR and resulted in 20 compounds (Hits). The 20 compounds typically consisted of all the inhibitory features possessed by the pharmL and pharmR as illustrated in Figure 5A. These compounds were additionally assessed for their behaviour at the proteins active site, employing the molecular docking simulations.

### 3.4. Molecular Docking-Based Screening

The resultant 20 compounds were upgraded to molecular docking studies to collectively refine the hit compounds and to discard the false positives. The suitability and the aptness of the docking parameters considered for both the docking programmes were assessed by docking the inbound ligand into the active site groove. This resulted in the generation of the pose with a binding mode as was observed within the crystal structure, with an acceptable RMSD of 0.97 Å for CDOCKER (Supplementary Figure S1A) and 1.25 Å for GOLD (Supplementary Figure S1B). Furthermore, among the 20 compounds, 14 compounds demonstrated the dock scores higher than the reference and the inbound ligand rendered by both the docking programmes and belonged to the largest cluster. These 14 compounds were examined for the knowledge-based screening by manually probing into their interactions with the key residues, yielding six compounds (Figure 5B, Supplementary Figure S2) from which the top three compounds were studied extensively.

### 3.5. Molecular Dynamics Simulations

**Figure 6:**
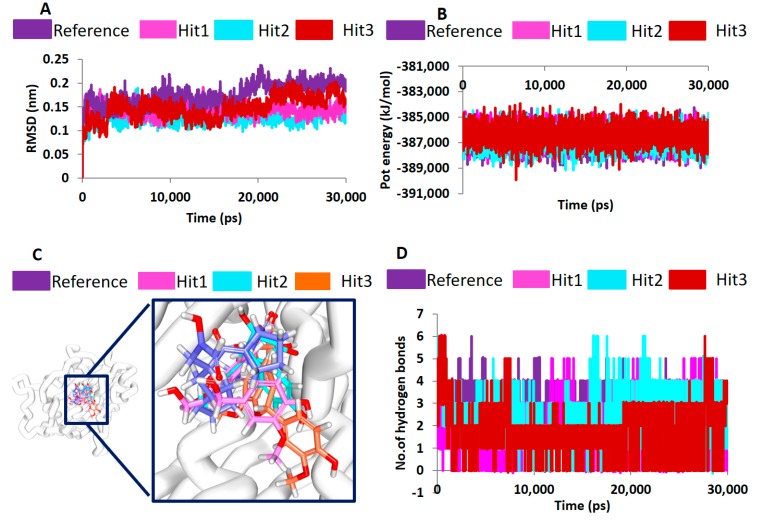
Molecular dynamics simulation results. (**A**) Stability assessment by root mean square deviation. (**B**) Stability assessment by potential energy profiles. Both the graphs demonstrate that the systems were highly stable through a 30-ns run. (**C**) Evaluation of the binding modes (**D**) Enumeration of stable H-bond interactions during 30-ns run.

MD studies have been extensively exploited to decipher the behaviour of the complex molecules at the atomic level. In the current study, the MD simulations were conducted to authenticate the docking results and binding stability and to affirm the binding modes by monitoring the orientation of the protein–ligand complex during 30 ns (Figure 5C). The best-docked poses obtained from the molecular docking results were taken as the initial structures for the MD. The findings were inferred as root mean square deviation (RMSD) of the protein backbone atoms, the potential energies for each system by interpreting the stability of the backbone atoms throughout the simulations, binding mode analysis, and hydrogen bond count (Figure 5C). The RMSD profiles of the three hits and reference were found to be below 2 nm throughout the simulations inferring that the systems were well converged. Upon examining the average RMSD of the reference, it was recorded to be 0.16 nm, while Hit1, Hit2, and Hit3 rendered values of 0.11 nm, 0.12 nm, and 0.12 nm, respectively (Figure 6A). Additionally, the potential energy profiles of the four systems remained relatively stable between −3.87× 10^5^ and ~−3.85× 10^5^ (Figure 6B). These results elucidate the stable nature of the systems during 30 ns.

Subsequently, the representative structures from the last 5 ns were extracted to delineate on the binding modes of the hits. Upon superimposition, it was found that the hits and the reference compounds were accommodated in the same binding pocket in a similar fashion, as was seen in the crystal structure anchored by the several key resides through hydrogen bonds, hydrophobic interactions, and the van der Waals interactions (Figure 6C).

**Figure 7:**
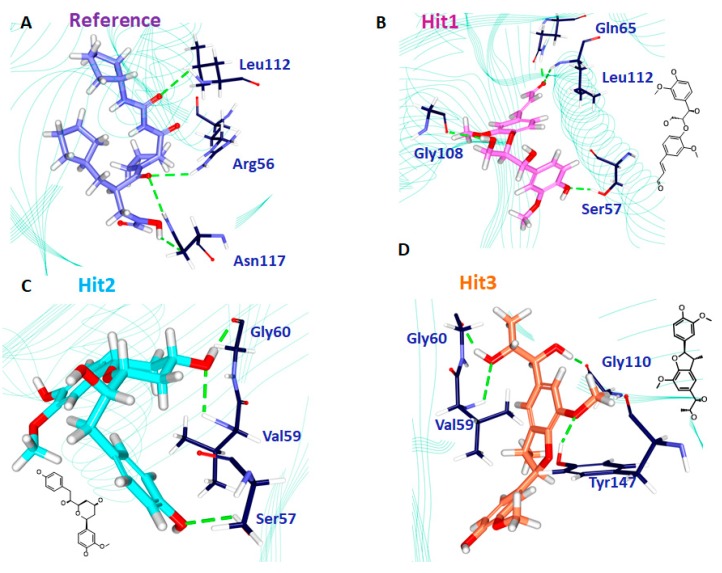
Intermolecular hydrogen bond interactions of the reference (**A**), Hit1 (**B**), Hit2 (**C**), and Hit3 (**D**), respectively.

Delineating on the interactions of the reference compound, it was revealed that the reference compound formed four hydrogen bonds involving three residues: Arg56, Leu112, and Asn117. The residue Asn117 demonstrated two hydrogen bonds with an acceptable bond length (Table 7 and Figure 7A). Furthermore, several hydrophobic charged residues interacted with the reference compound to accommodate the compound in an appropriate position in the proteins active site. The residues Ser57, Gly58, Gly60, Leu105, Gly108, Glu109, CDS111, Tyr147, and Glu155 held the reference compound firmly. Furthermore, the ring A interacted with the benzene ring of His154 by a π-alkyl interaction rendered by a bond length of 4.2 Å. The residues Val151 and Val59 formed alkyl hydrophobic interactions with ring A with a bond length of 4.2 Å and 5.1 Å, respectively. Additionally, Gly110 and Glu185 were noted to lock in the reference compound in the active site of the protein (Table 7). Moreover, the hits were observed to interact with different charged residues of the protein clamping the ligands at the active site of the proteins (Supplementary Figure S3).

Hit1 has formed four hydrogen bonds with the active site residues such as Ser57, Gln65, Gly108 and Leu112 with the bond length less <3 Å (Table 7 and Figure 7B). Additionally the residues Gly58, Val59, Leu61, Leu105, Thr107, Glu10, CSD111, Ile150, Val151, His154, and His186 aided in seating of the Hit1 at the active groove of the protein through van der Waals interactions. The residues Gly60, Glu108, Glu115, Tyr147, and Glu185 anchored to the ligand by forming the carbon-hydrogen bonds. Moreover, the O atom of Gly110 interacted with the ring B of Hit1 forming a π lone pair interaction rendered by a bond distance of 2.9 Å clamping the centre of the ligand. Such an interaction was not noticed with the other candidate compounds, portraying that it has a unique feature of Hit1 (Supplementary Figure S3). Careful assessment of the interaction reveals that the Ser57 and Gln65 andLeu112 have held the ligand towards the extreme ends while Gly108 was found to interact with the chain of the ligand. Additionally, the residues Gly58, Val59, Leu61, Leu105, Thr107, Glu109, CSD111, Ile150, Val151, His154, and His186 participated through van der Waals interaction, firmly accommodating the ligand at the active site (Supplementary Figure S3).

Hit2 generated three hydrogen bonds, one each with Ser57, val59, and Gly60, respectively showcased by an acceptable bond length (Table 7 and Figure 7C). The residues Gly110 and Glu185 were observed to form a carbon-hydrogen bonds while the residues Arg56, Gly58, Gln65, Leu61, Leu105, Glu109, Gly108, CDS111, Leu112, Arg124, Tyr147, Ile150, and Glu155 locked the ligand firmly through the van der Waals interactions. Furthermore, ring C of the ligand formed π/alkyl bonds with Val151 and His 154, respectively. The residue Val151 formed a hydrophophic π-alkyl bond with the ring C of the ligand with a bond length of 5.3 Å. The benzene ring of His154 residue has formed π-π stacked hydrophobic interaction with the C ring of the ligand portraying a bond distance of 4.1 Å. Deciphering of the distribution of the residue interactions, it was revealed that the Val151 and His 154 held one end of the ligand while the other end was held by the Ser57. Val59 and Gly60 residues clamped the centre portion of the ligand while the other charged atoms contribute to proper stationing of the ligand at the proteins active site, Supplementary Figure S3.

Hit3 promoted Val59, Gly60, Gly110, and Tyr147 hydrogen bonds, displaying by an acceptable bond length (Table 7 and Figure 7D). The residues Tyr147, Glu155, Glu185, and His186 involved in the formation of carbon hydrogen bonds. Additionally, several charged residues Leu41, Arg56, Ser57, Gly58, Leu61, Gln65, Pro78, Ile77, Glu109, CSD111, Gly110, Leu112, Ile150, His154, and His158 assisted in locking the ligand at the active site through the van der Waals interactions. Besides forming the hydrogen bond, the residue Val59 has additionally formed π-alkyl bonds. The ring A and ring B of the ligand has formed π-alkyl hydrophobic bonds displayed a bond length of 4.5 Å and 4.9 Å, respectively (Supplementary Figure S3). The residues Val59, Gly60, Gly110, and Tyr147 are distributed on either side of the ligand, while the charged residues served in proper lodging of Hit3 in the active site of the protein. From the obtained results, it can be drawn that the hits have prompted greater number of interactions upon comparing with reference compound. These findings are in parallel with the previously reported work [3]. The leads us to comprehend that the hits can offer better inhibitory effects than the reference compound and the cocrystal natural inhibitor actinonin.

### 3.6. Probing the Novelty of the Hits

The similarity search was performed employing the inbuilt *Find Similar Molecules by Fingerprints* tool within the DS using the well-defined parameters. The number of similar molecules was selected as five and the percentage of similar molecules was opted as one. The search was conducted employing the minimum similarity of 0.50 and 0.90, respectively. The similarity search analysis executed over three final Hits proved that they shared no similarity with the known PDF inhibitors. Furthermore, the SMILES-based ChemSpider search additionally reiterated the same illuminating that the hits were novel inhibitors of PDF.

## 4. Discussion

Bacterial peptide deformylase is an attractive target for developing new antibiotics because of its presence in prokaryotes and absence in eukaryotes [3]. Therefore, the current research focuses on identifying new inhibitors representing the inhibitory features of pharmL and pharmR, respectively. The pharmL was generated from the known PDF inhibitors, while pharmR was developed considering the available PDF targets and the innate ligands so as to generate a model representing the broad spectrum. Subsequently, these models retrieved a total of six compounds that were in common to both. This leads us to speculate that the prospective drug candidates have greater inhibitory potency towards the bacteria imbibed with the inhibitory features inherited from the ligand based and structure based pharmacophore models.

The hits demonstrated a higher number of molecular interactions than the reference compounds while been accommodated at the active site groove. Furthermore, the hydrogen bond interactions were tracked during the 30 ns simulation run. The results indicated that the hits have displayed greater number of hydrogen bonds as depicted in Figure 6D. Correspondingly, the average number of hydrogen bonds for reference and the hits were computed. The results exhibited that the reference had 1.8 hydrogen bonds, while the Hit1, Hit2, and Hit3 demonstrated an average of 2.0, 2.2, and 2.5 hydrogen bonds, respectively. These results are clear indicative that the hits have portrayed higher number of hydrogen bonds inferring the ability of the compounds as probable PDF inhibitors. Furthermore, upon scrupulous evaluation of the interactions, it was observed that the two residues, CDS111, and Glu185 have been consistent with the hits as was noticed with the reference anchored by the van der Waals interactions. These finding are in agreement with the previous reports [2] and further showcase the probable importance of these residues in inducing the inhibitory mechanism.

Furthermore, we meticulously contemplated on the accommodation of the reference and the hits at the active site of the protein. Structurally, the binding pocket of the PDF was further divided into three subsites, namely, S1’, S2’, and S3’, respectively [1]. The residues in vicinity to S1’ subunit of *Staphylococcus aureus* is comprised of Gly60, Leu105, Gly108, Glu109, Ile150, Val151, His154, and Glu155. The S2’ subunit was in proximity to Arg56, Cys111, and Leu112 while the S3’ subunit is encompassed of residues such as Ser57, Gly58, Val59, and Tyr147.

**Figure 8:**
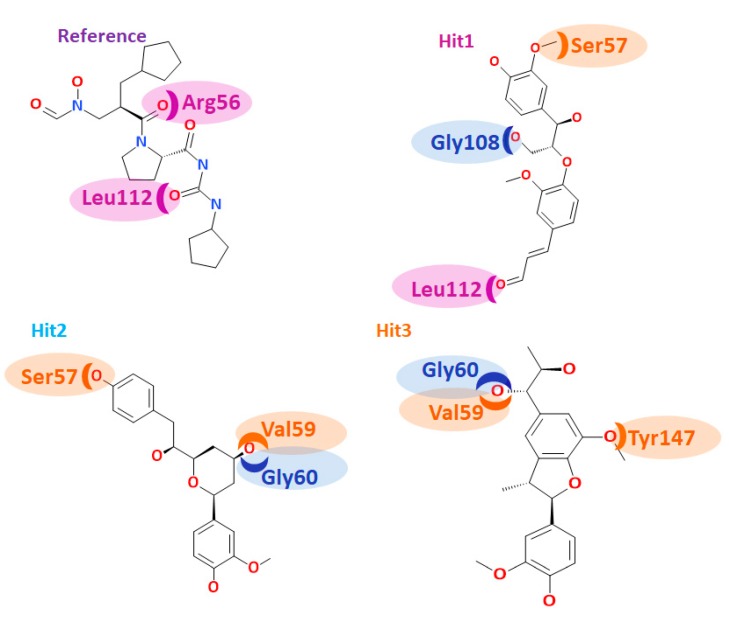
Subsite occupation of the compounds. The residues from S1’ region are represented in blue, the residues from S2’ region are indicated in purple and the residues from S3’ region are represented in orange colour, respectively.

The reference compound has formed hydrogen bond interactions with three residues (Table 7). The residues Arg56 and Leu112 belong to the proximity of subsite S2’, while the residue Asn117 lies in close proximity to subsite S2’ (Figure 8). The residues from the subsite S3’ Ser57, Gly58, and Tyr147 interacted with the ligand through the van der Waals interactions. The residues, Leu105, Gly108, Glu109, and Glu155 from the subsite S1’ also assisted to firmly nestle the reference compound at the active site. Furthermore, a rigorous evaluation of the hits revealed their interactions with the residues located at subsites S1’, S2’ and S3’. Hit1 interacted with Ser57 residue through hydrogen bond that hails from S3’, while the residue Gly108 belongs to S1’ subsite. The residue Leu112 that formed the hydrogen bond interaction with the ligand is contributed by the S2’ subsite of the binding pocket (Figure 8). The residues Gly60 and Glu115 that are originated from the S1’ subsite and Tyr147 from S3’ subsite firmly held the ligand through the carbon-hydrogen bond. Evaluating the van der Waals interactions, it was noted that the residues Gly58, Val59 are from the S3’ subsite, while the residues Leu105, Glu109, Ile150, Val151 and His154 from the S1’ subsite were found to interact with the van der Waals interactions. Hit2 rendered the hydrogen bond interactions with residues such as Ser57, Val59, and Gly60. The residues Ser57, Val59 were contributed by the S3’ subsite, while the residue Gly60 was from S1’ subsite, respectively (Figure 8). A single residue Val151 that anchored the ligand via alkyl/π- alkyl interactions is from S1’ subsite. From the residues that participated in the van der Waals interaction, Leu105, Gly108,Glu109,Ile150, and Glu155 are from S1’ subsite, Gly58, CDS111, and Leu112 reside at the S2’ subsite, and Gly58 and Tyr147 belong to the S’ subsite. Hit3 demonstrated the hydrogen bond interactions with Val59, Gly60, Gly110, and Tyr147 residues. The residue Val59 and Tyr147 originated from the S3’ subsite and Gly60 from the S1’ subsite, respectively (Figure 8). Additionally, the residues Glu155 from S1’ subsite and Tyr147 from S3’ subsite generated carbon–hydrogen bonds, while Val59 from S3’ subsite has formed alkyl/π-alkyl interactions. Moreover, the residues Glu109, Ile150, and His154 from S1’ subsite, residues Arg56, CDS111, and Leu112 from the S2’ subsite, and the residues Ser57 and Gly58 from the S3’ subsite displayed the van der Waals interactions with the ligand. Additionally, several other residues have been noted to favour the accommodation of the hits at the proteins active site (Supplementary Figure S3). These results guide us to infer that the hits nestled at the active site and were firmly seated through several key residues contributed by the three subsites.

**Figure 9:**
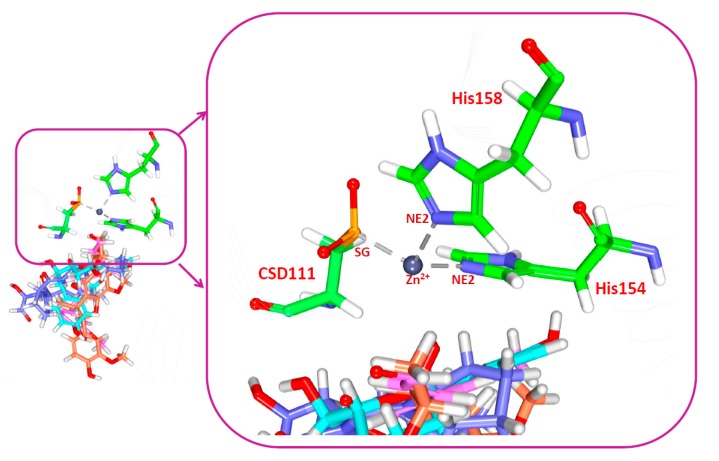
Stable triad metal ion interaction.

During the docking simulations, the bonded model of Zn^2+^ ion chelation was applied as reported previously [3] in which the coordinate bonds between the Zn^2+^ ion and the residues interacting with the protein were treated as covalent bonds. Moreover, the distance constraints for the three residues, CSD111, His154 and His158 that are involve in the Zn^2+^ ion were configured between 2.3–2.5 Å (Figure 9). The resultant molecular docking results have demonstrated a distance constraint within the fixed length without any variation. However, it is worth noting that the O23 atom of Hit1 has displayed an interaction with the metal ion, while Hit2 and Hit3 did not generate any interaction with the metal ion. Furthermore, it was observed that the hydrogen bonds of Hit1 were prompted from three subsites, thereby showing its significance as the most valuable antimicrobial agent (Figure 8). Taken together, the obtained results illuminate the importance of the hit compounds as potential PDF inhibitors. The three hits demonstrated higher dock scores than the reference compounds (Table 8), complemented by enhanced inhibitory activities. Additionally, the hits depicted all the features of the pharmL and pharmR, correspondingly (Supplementary Figure S4) and the two-dimensional structures are represented in Figure 5D. These findings suggest that the identified hits serve as potential broad spectrum antibiotic agents and further may serve as novel scaffolds in determining new drugs.

## 5. Conclusions

An ideal way to encounter MRDs is to use natural compounds due to their abundance and relatively low toxicity. In the current investigation a dual pharmacophore-based virtual screening method was executed to redeem the compounds using structure-based and ligand-based approaches. The ligand-based approach was carried out to redeem the compounds exploiting the chemical features from the known inhibitors, and the structure-based approach was performed to screen for compounds better than the inbound ligand. The computational approaches rendered 14 phytochemicals from the TIP database that demonstrated highest molecular dock scores and key residue interactions with the target protein. Upon subjecting the top three compounds to MD simulation studies, they displayed the stable RMSD values accommodated at the protein’s active site, demonstrating greater number of hydrogen bonds during the 30-ns MD run. The identified hits displayed stable interactions with the active site residues such as Ser57, Val59, Gly60, Gly110, and Leu112, respectively. Among the three identified compounds, Hit1 showed stable key interactions with respect to the three subsites. This compound also imbibes the features from pharmL and pharmR, illuminating its superiority as the most valuable antimicrobial agent. Taken together, we recommend three potential candidates as novel PDF inhibitors that can additionally serve as scaffolds in designing new lead candidates.

## Figures and Tables

**Table 1:** Statistical information of 10 pharmacophore hypotheses derived by HypoGen.

Hypo Number	Total Cost	Cost Difference ^a^	RMSD	Correlation	Features ^b^	Maximum Fit
Hypo1	127.67	113.10	1.77	0.90	2HBA, HBD, HyP	13.23
Hypo2	131.722	109.06	2.06	0.86	2HBA, HyP, HyP, HyP	13.34
Hypo3	132.165	108.62	1.94	0.88	2HBA, HBD, HyP	12.71
Hypo4	133.398	107.38	2.00	0.87	2HBA, HBD, HyP	12.27
Hypo5	133.808	106.97	2.14	0.85	2HBA, HyP, HyP, HyP	12.08
Hypo6	133.895	106.89	1.87	0.89	2HBA, HBD, HyP	13.88
Hypo7	135.009	105.77	2.09	0.86	2HBA, HBD, HyP	11.53
Hypo8	135.104	105.68	2.17	0.85	2HBD, HyP, HyP, HyP	12.29
Hypo9	135.444	105.34	1.97	0.88	2HBA, HBD, HyP	13.30
Hypo10	135.564	105.22	2.01	0.87	2HBA, HBD, HyP	12.91

^a^ Cost difference, difference between the null cost and the total cost. The null cost of 10 scored hypotheses is 240.78, the fixed cost value is 86.77, and the configuration cost is 18.37. All costs are represented in bit units. ^b^ HBA, hydrogen bond acceptor; HBD: hydrogen bond donor; HyP, hydrophobic; RMSD, root-mean-square deviation.

**Table 2:** Experimental and predicted activity values of training set compounds according to Hypo 1.

Name	Fit	IC_50_ (nmol/L)		RMSE ^a^	Activity Scale	
		Experimental	Predicted		Experimental	Predicted
C1	13.03	0.1	0.55	5.5	+++	+++
C2	12.29	0.3	3	10	+++	+++
C3	12.99	0.41	0.61	1.5	+++	+++
C4	12.98	0.5	0.62	1.2	+++	+++
C5	12.72	1	1.1	1.1	+++	+++
C6	12.54	2.1	1.7	−1.2	+++	+++
C7	12.72	8	1.1	−7	+++	+++
C8	11.44	15	22	1.4	+++	+++
C9	11.16	30	41	1.4	+++	+++
C10	10.24	52	350	6.6	+++	+++
C11	9.5	74	190	6	+++	+++
C12	9.46	300	2100	6.9	+++	++
C13	9.66	430	130	3	+++	+++
C14	9.32	800	2800	3.5	++	++
C15	9.61	3000	1400	−2.1	++	++
C16	9.97	7400	630	−12	++	++
C17	8.07	28,000	51,000	1.8	+	+
C18	9.65	54,000	1300	−40	+	++
C19	8.79	100,000	9600	−10	+	++
C20	8.8	560,000	9400	−6.0	+	++

^a^ RMSE, ratio of the predicted activity (Pred IC_50_) to the experimental activity (Exp IC_50_) or its negative inverse if the ratio is <1; IC_50_, half maximal inhibitory concentration; +, IC_50_ values are less than 100 nmol/L; +++, IC_50_ values existing between 100 nmol/L and ~10,000 nmol/L; (++),IC_50_ values greater than 10,000 nmol/L (+).

**Table 3:** Receptor-based pharmacophore generation.

Pharmacophore	Number of Features	Feature Set	Selectivity Score
Pharmacophore_1	6	HBA, HBD, HBD, HyP, HyP, HyP	11.498
Pharmacophore_2	6	HBA, HBD, HBD, HyP, HyP, HyP	11.498
Pharmacophore_3	6	HBA, HBD, HBD, HyP, HyP, HyP	11.498
Pharmacophore_4	6	HBA, HBD, HBD, HyP, HyP, HyP	11.498
Pharmacophore_5	6	HBA, HBD, HBD, HyP, HyP, HyP	11.498
Pharmacophore_6	6	HBA, HBD, HBD, HyP, HyP, HyP	11.498
Pharmacophore_7	6	HBA, HBD, HBD, HyP, HyP, HyP	11.498
Pharmacophore_8	6	HBA, HBD, HBD, HyP, HyP, HyP	11.498
Pharmacophore_9	6	HBA, HBD, HBD, HyP, HyP, HyP	11.498
Pharmacophore_10	6	HBA, HBD, HBD, HyP, HyP, HyP	11.498

**Table 4:** Root mean square deviation (RMSD) values generated through pharmacophore comparison between the ligand-based and the receptor-based models. Model 1 displayed the lowest RMSD (Å).

Model Number	RMSD
1	1.41
2	1.63
3	1.87
4	2.03
5	2.11
6	2.14
7	2.47
8	2.92
9	2.94
10	2.99

**Table 5:** Experimental and predicted activity values of test set compounds according Hypo1.

Name	Fit	IC_50_ (nmol/L)		RMSE ^a^	Activity Scale	
		Experimental	Predicted		Experimental	Predicted
C1	13.21	0.19	0.45	2.3	+++	+++
C2	13.21	0.19	0.45	2.3	+++	+++
C3	12.9	0.22	0.92	4.2	+++	+++
C4	13.07	0.31	0.61	2	+++	+++
C5	12.15	3	5.1	1.7	+++	+++
C6	13.07	4.4	0.62	−7.1	+++	+++
C7	11.54	7	21	3	+++	+++
C8	11.81	10	11	1.1	+++	+++
C9	10.95	16	81	5.1	+++	+++
C10	11.64	20	17	−1.2	+++	+++
C11	11.54	40	21	−1.9	+++	+++
C12	10.14	64	520	8.1	+++	+++
C13	10.81	100	98	1.1	+++	+++
C14	10.75	120	130	1.1	++	++
C15	10.02	170	690	4.1	++	++
C16	9.7	180	1400	8.2	++	++
C17	10.11	290	560	1.9	++	++
C18	9.43	330	2700	8.1	++	++
C19	10.01	590	700	1.2	++	++
C20	9.7	1000	1400	1.4	++	++
C21	9.86	1400	990	−1.4	++	++
C22	9.88	2200	950	−2.3	++	++
C23	8.89	4100	9200	2.2	++	++
C24	8.92	7500	8600	1.1	++	++
C25	8.39	21,000	29,000	1.4	+	+
C26	8.36	34,000	32,000	−1.1	+	+
C27	9.36	61,000	31,000	2.0	+	+
C28	9.48	80,000	24,000	3.4	+	+
C29	9.57	100,000	1900	−52	+	++
C30	8.95	200,000	8100	−25	+	++
C31	7.18	380,000	480,000	1.3	+	+

^a^ RMSE, ratio of the predicted activity (Pred IC_50_) to the experimental activity (Exp IC_50_) or its negative inverse if the ratio is <1.

**Table 6:** Decoy set validation of both the pharmacophore models.

Parameters	PharmL	PharmR
Total number of molecules in database (D)	1000	1000
Total number of actives in database (A)	20	20
Total number of hit molecules (Ht)	25	24
Total number of active molecules (Ha)	19	20
% Yield of active ((Ha/Ht) × 100)	76.0	83.3
% Ratio of actives ((Ha/A) × 100)	95	100
Enrichment factor (EF)	38.0	41.5
False negatives (A-Ha)	1	0
False positives (Ht–Ha)	5	4
Goodness of fit score (GF)	0.79	0.83

D, external dataset; A, active compounds; Ht, Total number of hit compounds; Ha, total number of active compounds; EF, enrichment factor; GF, goodness of fit.

**Table 7:** Intermolecular molecular interactions between the proteins and the hits.

Name	Hydrogen Bond (<3 Å)	Alkyl/π- alkyl	Van der Waals Interactions
Ref	Arg56: HH21-O5 (2.7) Leu112: HN-O24 (2.9) Asn117: HD22-O5 (2.8) Asn117: OD1-H35 (2.1)	Val59, Val151, His159	Ser57, Gly58, Gly60, Leu105, Gly108, Glu109, CDS111, Tyr147, Glu155, Glu185
Hit1	Ser57: HG-O17 (2.1) Gln65: HE22-O23 (1.8) Gly108: O-H49 (2.4) Leu112: HN-O23 (2.6)	-	Gly58, Val59, Leu61, Leu105, Thr107, Glu109, CSD111, Ile150, Val151, His154, Glu185 His186
Hit2	Ser57: HG-O16 (2.7) Val59: HN1-O23 (1.8) Gly60: O-H44 (1.7)	Val151	Arg56, Gly58, Gln65, Leu61, Leu105, Gly108, Glu109, CDS111, Leu112, Arg124, Tyr147, Ile150, Glu155, Glu185
Hit3	Val59: HN-O22 (2.9) Gly60: O-H50 (1.8) Gly110: O-H46 (1.8) Tyr147: HH-O1 (2.1)	Val59	Leu41, Arg56, Ser57, Gly58, Leu61, Gln65, Pro78, Ile77, Glu109, Gly110, CSD111, Leu112, Ile150, His154, His158, Glu185

**Table 8:** Tabulation of the dock scores of reference and the hits employing two different approaches.

Compound Name	-CDOCKER Energy	-CDOCKER Interaction Energy	GoldScore	ChemScore
Reference1	4.00	46.42	42.86	–19.68
Actinonin	29.12	45.94	41.05	–18.56
Hit1	30.66	51.11	51.29	–26.55
Hit2	30.76	50.13	48.55	–27.00
Hit3	21.78	47.32	55.33	–28.97

## Supplementary Materials

The following are available online at http://www.mdpi.com/2077-0383/7/12/563/s1, Figure S1: Evaluation of the docking parameters; Figure S2: 2D structures of six compounds; Figure S3: Intermolecular interactions; Figure S4: Pharmacophore models and hits alignment.
