# Supplementary material for: Discovery of Potential Plant-Derived Peptide Deformylase (PDF) Inhibitors for Multidrug-Resistant Bacteria Using Computational Studies
Source: J Clin Med. 2018 Dec 17;7(12):563. doi: 10.3390/jcm7120563 (PMC6306950; doi:10.3390/jcm7120563)
Supplement: Supplementary file 1 [file jcm-07-00563-s001.pdf]

A

Docking of cocrystal into the proteins active site employing CDOCKER

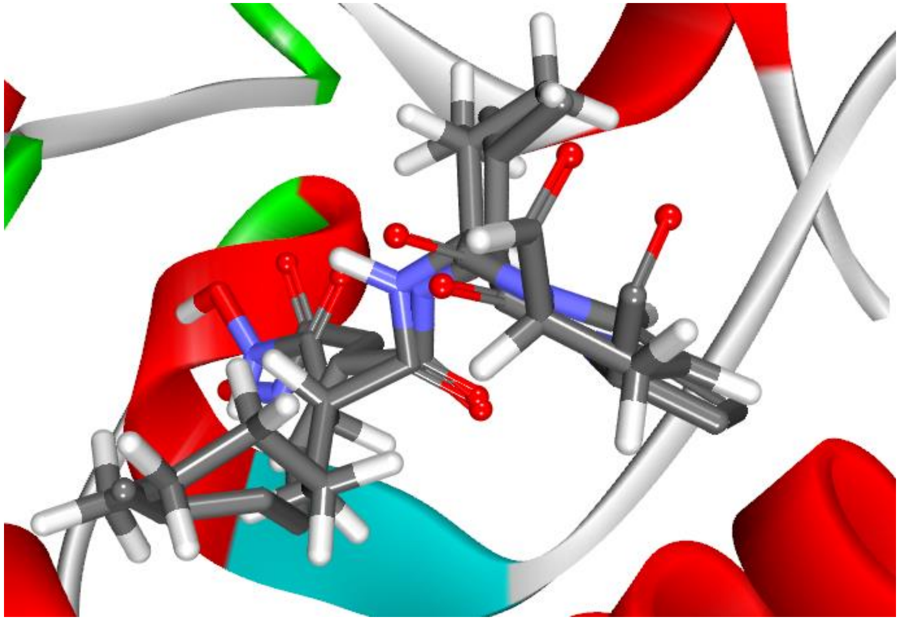

Docking of cocrystal into the proteins active site employing GOLD

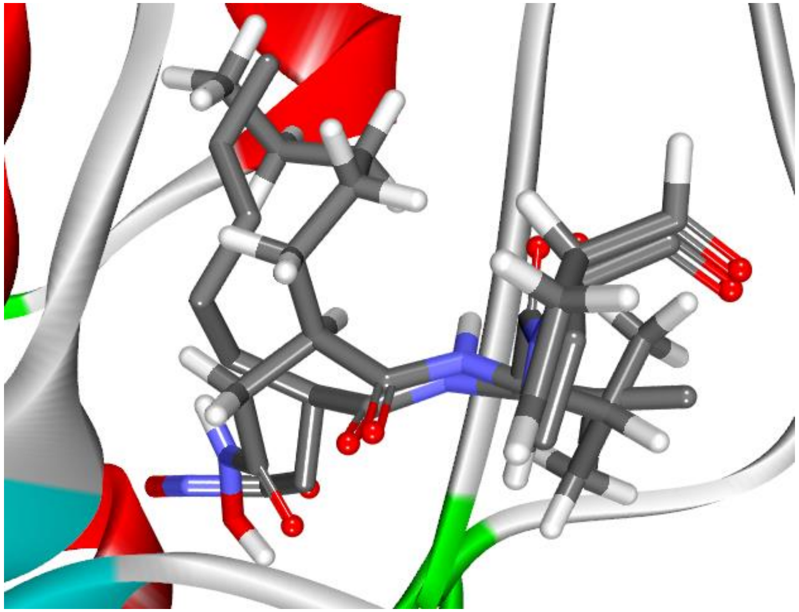

Supplementary Figure 1. Evaluation of the docking parameters

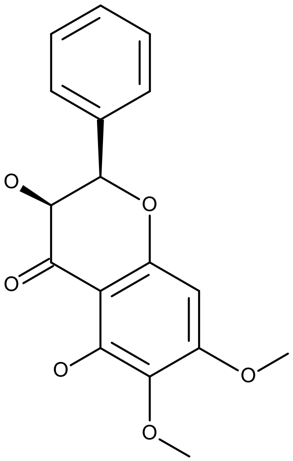

Compound 1

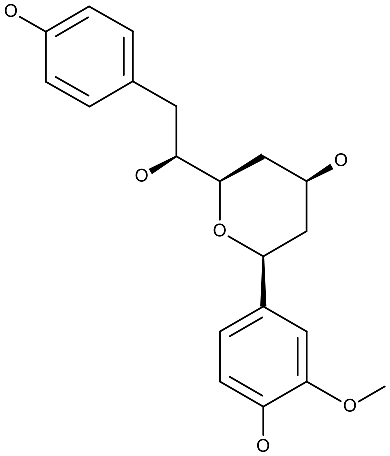

Compound 2

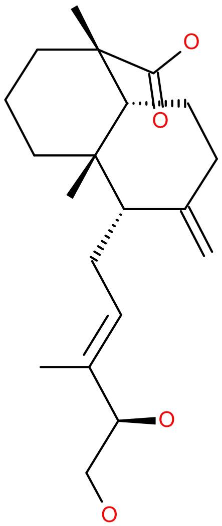

Compound 3

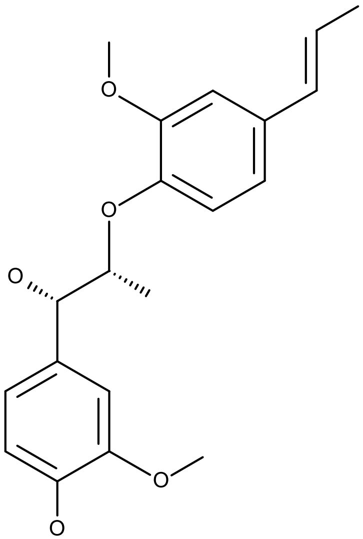

Compound 4

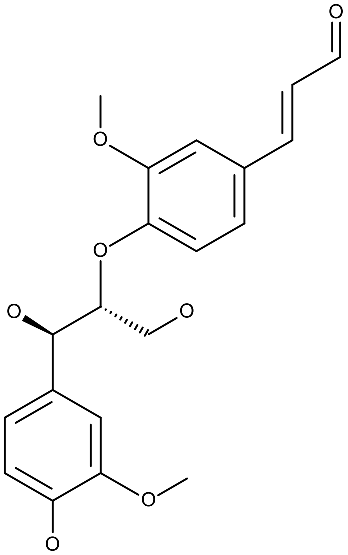

Compound 5

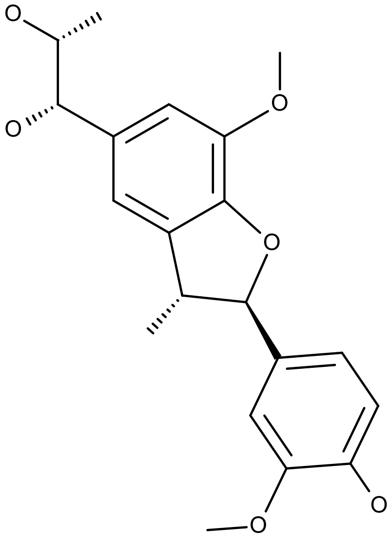

Compound 6

Supplementary Figure 2. 2D structures of six compounds

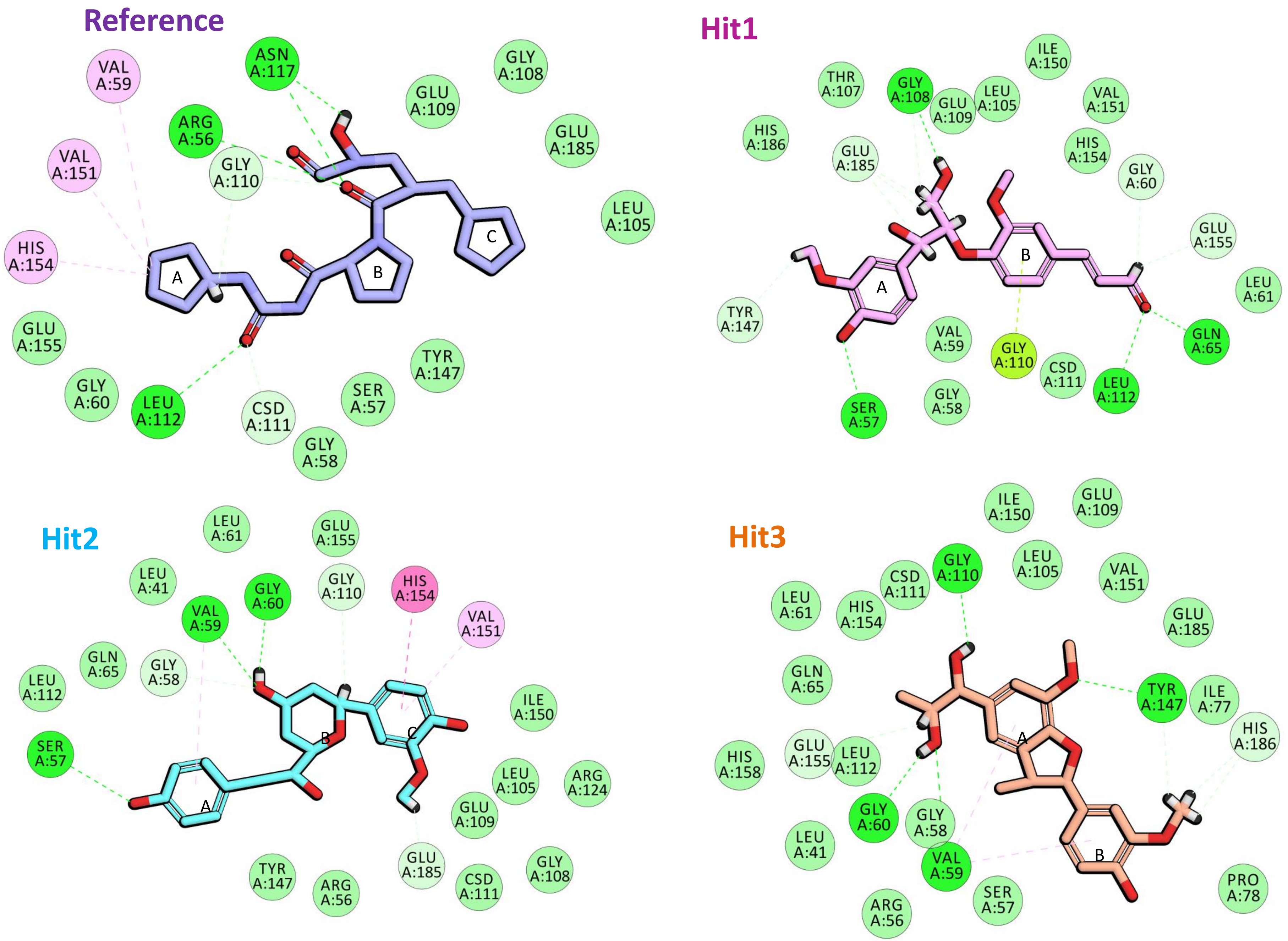

Supplementary Figure 3. Intermolecular interactions

Ligand-based pharmacophore mapping to the compounds

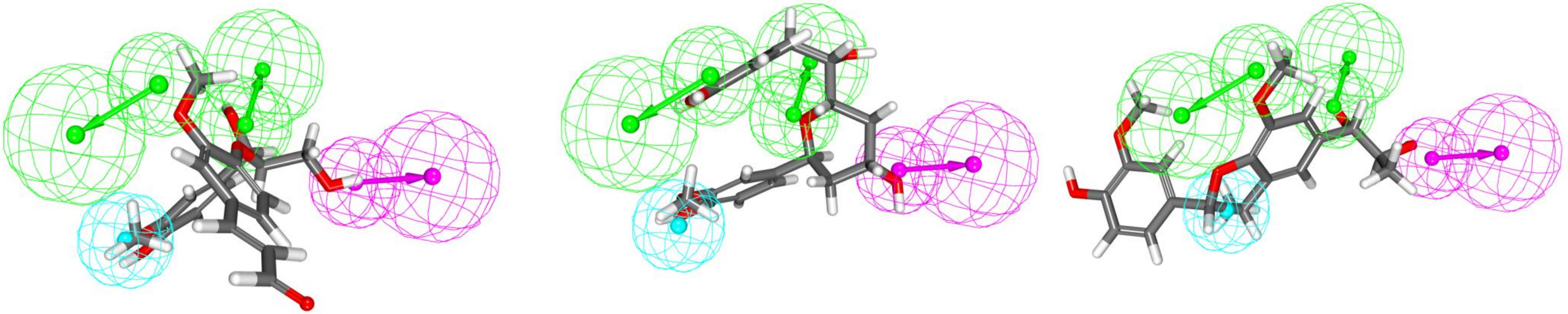

Structure-based pharmacophore mapping to the compounds

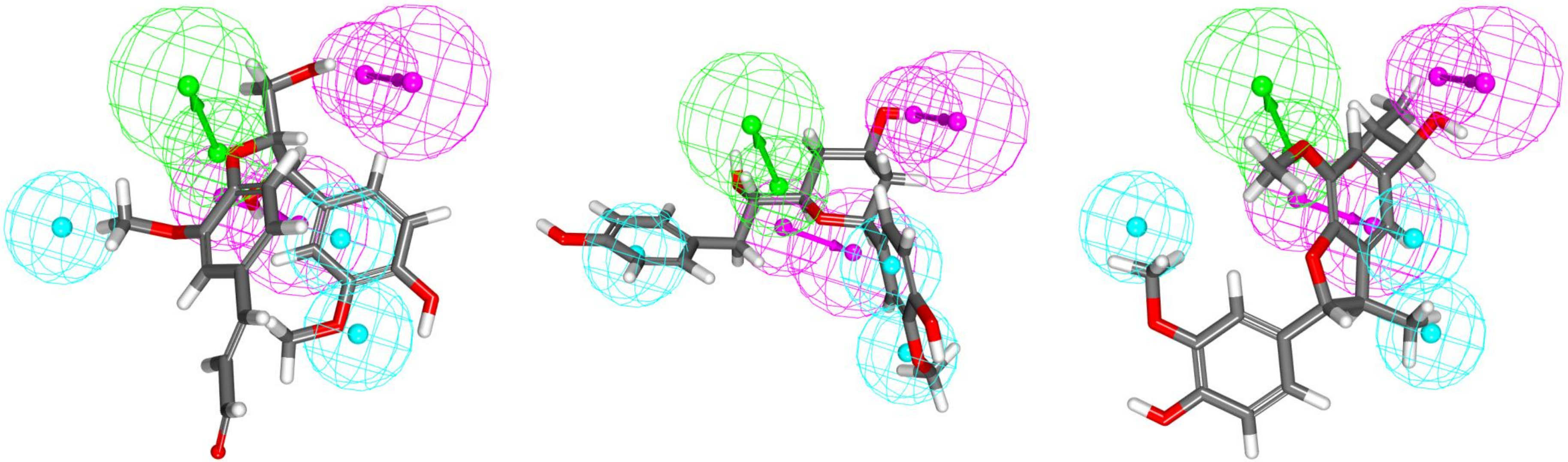

Supplementary Figure 4. Pharmacophore models and hits alignment
